# Supplementary material for: Dynalign II: common secondary structure prediction for RNA homologs with domain insertions
Source: Nucleic Acids Res. 2014 Nov 21;42(22):13939–48. doi: 10.1093/nar/gku1172 (PMC4267632; doi:10.1093/nar/gku1172)
Supplement: SUPPLEMENTARY DATA [file supp_gku1172_nar-02021-z-2014-File012.zip › manual/RNA_class/html/functions_0x73.html]

RNAstructure Classes: Class Members

- Main Page
- Classes
- Files

- Class List
- Class Hierarchy
- Class Members

- All
- Functions
- Variables
- Typedefs

- a
- b
- c
- d
- e
- f
- g
- h
- i
- l
- m
- n
- o
- p
- q
- r
- s
- t
- v
- w
- ~

Here is a list of all class members with links to the classes they belong to:

### - s -

- savefileread
  : Dynalign\_object- seqPair
    : Multilign\_object- sequences
      : HybridRNA- SetExtrinsic()
        : RNA- SetForbidIntramolecular()
          : HybridRNA- SetIndexSeq()
            : Multilign\_object- SetIterations()
              : Multilign\_object- SetMaxDsv()
                : Multilign\_object- SetMaxPairs()
                  : Multilign\_object- SetModifier()
                    : RNA- SetNucType()
                      : Multilign\_object- SetProgress()
                        : RNA
                        , Dynalign\_object
                        , HybridRNA
                        , Multilign\_object- SetSHAPEIntercept()
                          : Multilign\_object- SetSHAPESlope()
                            : Multilign\_object- SetTemperature()
                              : Multilign\_object
                              , Thermodynamics
                              , TwoRNA- SetupBimolecular()
                                : HybridRNA- SHAPEIntercept
                                  : Multilign\_object- SHAPESlope
                                    : Multilign\_object- single\_z\_predict()
                                      : Multifind\_object- SpecifyPair()
                                        : RNA- Stochastic()
                                          : RNA- StopProgress()
                                            : Multilign\_object
                                            , RNA
                                            , Dynalign\_object
                                            , HybridRNA- storetemplatefilename()
                                              : Dynalign\_object- structurecoordinates
                                                : RNA- sum\_multifind()
                                                  : Multifind\_object

---

Generated on Tue Jul 30 10:52:29 2013 for RNAstructure Classes by 
 1.5.7.1
